# Beyond the evidence: treating pulmonary hypertension in the intensive care unit

**DOI:** 10.1186/s13054-014-0524-y

**Published:** 2014-10-16

**Authors:** Seth R Bauer, Adriano R Tonelli

**Affiliations:** Department of Pharmacy, Cleveland Clinic, 9500 Euclid Avenue, Hb-105, Cleveland, OH 44195 USA; Respiratory Institute, Cleveland Clinic, 9500 Euclid Avenue, A-90, Cleveland, OH 44195 USA

## Abstract

Most patients with pulmonary arterial hypertension succumb to their disease in the ICU; however, limited evidence-based information exists to guide treatment in those that present with advanced right ventricular failure. Critical care physicians should be aware of the complexities of the treatment of patients with pulmonary arterial hypertension and should develop a strategy for their care. Current management is based on the pathophysiology of the disease and involves a multidisciplinary team supported by institutional polices directed at optimizing patient safety.

The article by Muzevich and colleagues addresses important issues surrounding medication therapy in patients with pulmonary arterial hypertension (PAH) that present to the ICU [1]. This review is very relevant to the critical care physician since most patients with PAH die from their underlying disease in the ICU while receiving PAH-specific therapy [2]. Fortunately, there are now nine pharmacological agents with US Food and Drug Administration-approved labeling for use in PAH, two of which were approved in 2013. These medications have marked pharmacological differences, which poses a particular challenge in critical care patients. Muzevich and colleagues have summarized the literature and presented their experience in the management of PAH-specific therapies in ICU patients [1]. We have selected particularly challenging areas to emphasize important points, present an alternative approach and identify areas for future research.

Of particular relevance is the lack of data on the comparative effectiveness of different vasodilator therapies either used alone or in combination [3]. Further research in this area is certainly needed. Parenteral prostacyclin analogs (that is, epoprostenol and treprostinil) are recommended for patients with PAH who present with right heart failure or progression of their disease [4]. However, the best treatment approach is unclear for patients with PAH and worsening right heart failure while already receiving a parenteral prostacyclin analog. In these patients, we continue all PAH-specific therapies unless there is a suspicion that the patient’s presentation could be a side effect of any of these medications (particularly in cases of recent dose changes or initiation of therapy). Two noteworthy side effects are lower extremity edema in patients treated with endothelin receptor antagonists [5] and pulmonary edema in patients with pulmonary veno-occlusive disease treated with parenteral prostacyclin analogs [6]. Pulmonary veno-occlusive disease is a rare form of pulmonary hypertension that is particularly challenging to diagnose and is characterized by narrowing of the small pulmonary veins.

In PAH patients with worsening right heart failure, we increase the dose of the prostacyclin analog and, if they are not already on this treatment, we start oral sildenafil (initial dose 10 mg every 8 hours with uptitration as tolerated). Sildenafil, a well-tolerated phosphodiesterase-5 inhibitor, is the preferred treatment due to its rapid onset of action and a relatively short plasma half-life (approximately 4 hours). Conversely, endothelin receptor antagonists require weeks to demonstrate noticeable pharmacodynamic effects.

If the cardiac index is low, we add dobutamine or milrinone since these inotropic agents have vasodilatory properties, thereby increasing right ventricular function and reducing pulmonary vascular resistance [7]. In patients receiving treprostinil subcutaneously we switch this prostacyclin analog to intravenous administration, given that the absorption of treprostinil via the subcutaneous route might not be reliable in this particular situation [8]. We use aerosolized epoprostenol in severely ill patients who do not tolerate rapid titration of intravenous prostacyclin analogs or who have hypoxemia not explained by a right-to-left shunt. Of note, we utilize the epoprostenol formulation that contains glycine (Flolan®; GlaxoSmithKline, Research Triangle Park, NC, USA) instead of the formulation containing arginine (Veletri®; Actelion, South San Francisco, CA, USA) because of the more extensive safety data, particularly in postoperative patients [9]. We have noted many technical challenges with the administration of aerosolized epoprostenol (such as cannula displacement and interruptions in the delivery system) leading to unexpected hemodynamic decompensation [10,11]. Extensive staff training and procedures to ensure safe use are therefore of the outmost importance when using aerosolized epoprostenol.

Catheter-related bloodstream infection is not uncommon in patients with PAH receiving a parenteral prostacyclin, with an incidence of approximately 0.3 to 0.5 cases per 1,000 treatment-days [12,13]. In our institution, patients presenting with a catheter-related bloodstream infection have a peripheral intravenous line placed and the tunneled catheter is rapidly removed. Our nurses are trained to obtain a reliable peripheral intravenous line and to check its status on a regular basis. For patients in whom a reliable peripheral venous access cannot be established, a peripheral inserted central catheter is placed for parenteral prostacyclin administration. Once clinical defervescence occurs and blood cultures remain negative for ≥2 days (other centers recommend ≥4 days [14]), a new tunneled catheter is inserted. This approach has allowed us to decrease the hospital length of stay. The Pulmonary Hypertension Association has made important recommendations to prevent central venous catheter infections in PAH receiving parenteral prostacyclin analogs [14]. These recommendations include the use of a cuffed and tunneled catheter with the minimum number of lumens (ideally one) and a closed hub system.

Given that the treatments for patients with PAH are complex and mistakes are possible, it is important to emphasize the need for a multidisciplinary team approach that includes critical care physicians, pulmonary hypertension experts, pharmacists, experienced nurses and social workers. In addition, there should be institutionally tailored protocols for the use of PAH therapies to prevent errors during the prescription, preparation or administration of these agents. These protocols could be accomplished by the use of order templates and extensive pharmacy and nursing checks.

## Abbreviation

PAH: pulmonary arterial hypertension
